# The effect of intellectual ability on functional activation in a neurodevelopmental disorder: preliminary evidence from multiple fMRI studies in Williams syndrome

**DOI:** 10.1186/1866-1955-4-24

**Published:** 2012-10-26

**Authors:** Jennifer R Pryweller, Suzanne N Avery, Jennifer U Blackford, Elisabeth M Dykens, Tricia A Thornton-Wells

**Affiliations:** 1 Interdisciplinary Studies in Neuroimaging of Neurodevelopmental Disorders, Vanderbilt University Medical Center, Nashville, TN, USA; 2 Vanderbilt Kennedy Center for Research on Human Development, Vanderbilt University, Nashville, TN, USA; 3 Vanderbilt Brain Institute, Neuroscience Graduate Program, Vanderbilt University, Nashville, TN, USA; 4 Department of Psychology, Vanderbilt University, Nashville, TN, USA; 5 Department of Psychiatry, Vanderbilt University School of Medicine, Nashville, TN, USA; 6 Department of Psychology and Human Development, Peabody College|, Vanderbilt University, Nashville, TN, USA; 7 Center for Human Genetics Research, Department of Molecular Physiology and Biophysics, Vanderbilt Institute of Imaging Science, Vanderbilt University Medical Center, Nashville, TN, USA

**Keywords:** IQ, BOLD fMRI, Williams syndrome, Intellectual disability

## Abstract

**Background:**

Williams syndrome (WS) is a rare genetic disorder caused by the deletion of approximately 25 genes at 7q11.23 that involves mild to moderate intellectual disability (ID). When using functional magnetic resonance imaging (fMRI) to compare individuals with ID to typically developing individuals, there is a possibility that differences in IQ contribute to between-group differences in BOLD signal. If IQ is correlated with BOLD signal, then group-level analyses should adjust for IQ, or else IQ should be matched between groups. If, however, IQ is not correlated with BOLD signal, no such adjustment or criteria for matching (and exclusion) based on IQ is necessary.

**Methods:**

In this study, we aimed to test this hypothesis systematically using four extant fMRI datasets in WS. Participants included 29 adult subjects with WS (17 men) demonstrating a wide range of standardized IQ scores (composite IQ mean = 67, SD = 17.2). We extracted average BOLD activation for both cognitive and task-specific anatomically defined regions of interest (ROIs) in each individual and correlated BOLD with composite IQ scores, verbal IQ scores and non-verbal IQ scores in Spearman rank correlation tests.

**Results:**

Of the 312 correlations performed, only six correlations (2%) in four ROIs reached statistical significance at a *P* value < 0.01, but none survived correction for multiple testing. All six correlations were positive. Therefore, none supports the hypothesis that IQ is negatively correlated with BOLD response.

**Conclusions:**

These data suggest that the inclusion of subjects with below normal IQ does not introduce a confounding factor, at least for some types of fMRI studies with low cognitive load. By including subjects who are representative of IQ range for the targeted disorder, findings are more likely to generalize to that population.

## Background

Williams syndrome (OMIM#194050) is a rare neurodevelopmental disorder caused by a hemizygous microdeletion on chromosome 7 (7q11.23), involving approximately 25 genes [1,2]. The neurocognitive profile of individuals with Williams syndrome (WS) is characterized by weakness in visuospatial construction alongside relative strengths in verbal short-term memory, expressive language, and face processing [3-6]. The personality profile of individuals with WS is marked by non-social anxiety and fears, hypersociability, and heightened empathy [7-11]. Individuals with WS frequently demonstrate a strong attraction to music and a fascination with certain sounds, paired with auditory hypersensitivity (hyperacusis) and phonophobia [12-17]. It has been suggested that maladaptive emotional and behavioral responses to environmental stimuli in persons with WS might be related to increased non-social anxiety, fear, and arousal or to sensory modulation impairments [8,18-20]. Functional neuroimaging studies in WS have aimed to investigate several aspects of the WS phenotype.

Although there is considerable inter-individual variability, most studies indicate mild to moderate intellectual disability in individuals with WS, reporting a range of IQ scores from 40 to 100, with a mean between 50 and 60 (see Table 1) [21-24]. Though the significance and size of the difference is still debated, studies consistently find verbal IQ is greater than non-verbal IQ in WS [22,24-26], which is consistent with the neurocognitive profile [4,23,27]. When using functional magnetic resonance imaging (fMRI) to compare individuals with ID to typically developing individuals, there is a possibility that differences in IQ contribute to between-group differences in blood oxygen level-dependent (BOLD) signal. If IQ were correlated with BOLD signal, then group-level analyses should adjust for IQ, or else IQ should be matched between groups. If, however, IQ is not correlated with BOLD signal, no such adjustment or criteria for matching (and exclusion) based on IQ is necessary.

**Table 1:** Intellectual ability in Williams syndrome

**Author (year)**	***n***	**Age (years)**	**Measure**	**FSIQ**		**VIQ**		**PIQ**	
				**Mean**	**(Range)**	**Mean**	**(Range)**	**Mean**	**(Range)**
Boddaert *et al.* (2006)	9	5-15	WISC-III	63	(50–79)	76	(62–91)	53	(43–65)
Don *et al*. (1999)	18	8-13	WISC-III	52	nr	61	(46–81)	50	(45–62)
Howlin *et al*. (1998)	62	19-39	WAIS-R	60	nr	64	nr	60	nr
Pagon *et al*. (1987)	9	10-20	WISC-R	42	(40–75)	54	(45–85)	52	(45–69)
Reiss *et al*. (2004)	43	12-50	WISC-R, WAIS-R	68	(46–83)	nr	nr	nr	nr
Searcy *et al*. (2004)	80	17-52	WAIS-R	67	nr	71	nr	66	nr

For each study, participant ages, IQ measure, full scale IQ (FSIQ), verbal IQ (VIQ), and performance IQ (PIQ) are listed. nr, not reported.

To date, approximately 18 published functional magnetic resonance imaging (fMRI) studies compare a WS sample to typically developing controls [28-45]. Six of the 18 studies restricted their recruitment of participants with WS to those who had an IQ within approximately one standard deviation of normal for the general population (see Table 2) [31-33,38,40,42]. With a mean IQ of 60, only 4.5% of those with WS have an IQ in this range, leading one-third of published studies to exclude a representative 95.5% of the WS population. This raises concerns about the generalizability of study findings to the majority of people with WS. Although some of the studies using this exclusion criterion were focused on using WS as a model to understand a particular aspect of the WS neurocognitive profile, at a minimum, this highlights a gap in research that is aimed at understanding WS *per se* as opposed to only specific components of the syndrome. In this study, we aimed to examine the relationship between IQ and BOLD activation using four extant fMRI datasets in WS. We hypothesized that in task-related studies with low cognitive load, IQ would not be correlated with BOLD activation.

**Table 2:** WS fMRI studies using low IQ as exclusion criterion for WS participants

**Author (year)**	**fMRI task(s)**	**Group**	***n***	**Sex**	**Age (SD)**	**IQ measure**	**IQ composite mean (SD)**
Meyer-Lindenberg *et al*. (2004)	Visual processing tasks (1. Passive viewing; 2. Visuospatial matching/constructive; 3.Visual attention)	WS	13^a^	6 M, 7 F	28.3 (9.6)	WASI (short form)	92.1 (9.6)
		CTL	11	6 M, 5 F	30.8 (7.6)	WAIS-R (short form)	96.8 (6.5)
Meyer-Lindenberg *et al*. (2005a)	Visual matching (stimuli = faces, scenes, shapes)	WS	9	3 M, 6 F	31.6 (9.6)	nr	92.4 (7.8)
		CTL	10	6 M, 4 F	29.0 (4.9)	nr	97.5 (6.4)
Meyer-Lindenberg *et al*. (2005b)	Passive viewing (stimuli = faces, houses, scrambled)	WS	13^a^	6 M, 7 F	28.3 (9.6)	WASI (short form)	92.1 (9.6)
		CTL	11^b^	6 M, 7 F	28.3 (9.6)	WAIS-R (short form)	97.9 (7.6)
Muñoz *et al*. (2010)	Visual/Emotional processing tasks (1. Emotional content labeling; 2. Scenes matching; 3. Shape matching)	WS	13^a^	6 M, 7 F	28.3 (9.62)	WASI (short form)	92.1 (9.6)
		CTL	11**	6 M, 5 F	30.8 (7.6)	WAIS-R (short form)	97.9 (7.6)
Olsen *et al*. (2009)	Passive viewing (stimuli = checkerboard, expanding ring; with button press)	WS	10	5 M, 5 F	31.3 (9.0)	WASI (short form)	92.1 (8.8)
		CTL	10	3 M, 7 F	29.3 (5.0)	WAIS-R (short form)	96.2 (7.4)
Sarpal *et al*. (2008)	Passive viewing (stimuli = faces, houses, scrambled)	WS	9	6 F, 3 M	31.6 (nr)	nr	92.4 (nr)
		CTL	10	4 M, 6 F	29 (nr)	nr	97.5 (nr)

Eighteen Williams syndrome (WS) fMRI studies use typically developing controls (CTL) as a contrast group. These six of the 18 studies exclude WS participants with low IQ.

^a^The same group of WS participants was used in these studies.

^b^The same group of control participants was used in these studies.

nr, not reported; WAIS-R, Wechsler Adult Intelligence Scale - Revised (Wechsler, 1981); WASI, Wechsler Abbreviated Scale of Intelligence (Wechsler, 1999).

## Methods

### Subjects

The extant datasets used for this analysis were drawn from four small fMRI studies conducted over a 6-year period. Participants included 29 individuals with WS (17 men, three left-handed) aged 16 to 59 years (mean = 25.2, SD = 8.4) recruited from the annual Vanderbilt Kennedy Center’s Williams Syndrome Music Camp, sponsored by the Vanderbilt Kennedy Center for Research on Human Development. Because scan acquisition was ongoing over several years, some campers participated in more than one fMRI study (see Additional file 1 Table S1 for participant enrollment by study).

We used multiple techniques in an effort to minimize participant anxiety about the MRI scan. To avoid anxiety related to the unfamiliar sounds they would hear during their MRI, an audio CD was recorded with the full-length sound that each imaging sequence would make during the scan session, and a copy of the CD was sent to each new participant prior to his or her attendance at Music Camp. During camp, participants interacted with imaging research staff, and prior to their actual scan, they were exposed to a mock MRI scanner that looked identical to the real one but did not contain the magnet. They were given the opportunity to lie down on the scanner bed and listen to the scanner sounds again. We also employed one participant with WS as a peer mentor. This mentor had successfully completed MRI scans with us in previous years and could to talk to his peers about his experience. Each participant gave his or her informed assent, and the participant’s parent or guardian gave informed consent prior to each study. Study protocols were approved by the Vanderbilt University Medical Center Institutional Review Board.

### Intellectual assessment

The *Kaufman Brief Intelligence Test, Second Edition* (KBIT-2), a brief measure of verbal and non-verbal intelligence, was administered to each participant. Standard scores (typical population: mean = 100, SD = 15) for verbal and non-verbal domains, as well as an IQ composite, are obtained. The KBIT-2 was developed for research or screening purposes in persons aged 4 years through adulthood. K-BIT2 scores correlate highly with other IQ tests and have been used successfully in WS and other samples with IDD [46,47]. Administration is brief, accommodating a population that presents functional or behavioral challenges that would otherwise preclude the use of a longer intellectual assessment.

### Data acquisition

Functional MR images (time of repetition (TR) = 2,000 ms, time to encode (TE) = 35 ms, flip angle = 79°, 3.5-mm slice thickness with a 0.35-mm gap, 240 mm^2^ field of view, 1.875 × 1.875 × 3.85 mm^3^ voxel size, sensitivity encoding (SENSE) factor = 1.5) were acquired using a single-shot T2-weighted gradient-echo echo-planar sequence, sensitive to changes in BOLD contrast. Slices were acquired parallel to the anterior-posterior commissural line (AC-PC) with an 80 × 80 pixel image matrix, reconstructed to 128 × 128 pixels. The number of functional MR image slices in each volume varied slightly by task-related study (as described below): Aud-MNS (33 slices), Music-Noise (31 slices), Faces (32 slices), Images (32 slices). High-resolution T1-weighted (T1W) anatomical volume images were acquired at the same location (TR = 4.6 ms, TE = 9 ms, 256 mm^2^ field of view, 1 × 1 × 1 mm^3^ voxel size, 170 sagittal slices). All images were obtained using a Philips Achieva 3-Tesla MRI scanner (Philips Healthcare, Inc., Best, The Netherlands).

Visual stimuli for fMRI tasks were rear-projected onto a translucent screen placed above the head coil and viewed through a double mirror attached to the head coil. Binaural auditory stimuli were delivered through the scanner’s pneumatic auditory stimulation system via standard Philips headphones. The presentation of audio-visual stimuli, controlled through E-Prime Software (Psychological Software Tools, Pittsburgh, PA, USA), was synchronized with data acquisition by a trigger pulse delivered by the scanner console.

### Study design and stimuli

The extant datasets used for this analysis were drawn from four smaller fMRI studies conducted over a 6-year period.

#### Auditory mirror neuron system study (Aud-MNS)

We obtained stereophonic sounds from the International Affective Digital Sounds (IADS) database, and we selected 20 sounds for auditory stimuli, which we classified into one of four groups: hand actions, mouth actions, laughter, or environment. Hand actions, mouth actions, and laughter sounds were selected as stimuli likely to activate the human mirror neuron system (MNS), and environmental sounds were selected as stimuli that were unlikely to activate the MNS. To avoid misinterpretation of auditory stimuli, participants were familiarized with the sounds during a practice task prior to the scan. The practice task consisted of presenting each of the auditory stimuli on a computer while the participant viewed a white fixation cross on a black screen. Following two successive presentations of each sound, the fixation cross disappeared, and a description of the stimulus sound was displayed on the screen. The description of each sound was read to the participant out loud.

The Aud-MNS study employed a block design with four passive-listening stimulus conditions: hand action sounds, mouth action sounds, laughter sounds, and environmental sounds, plus a silent/rest condition. Each stimulus block was comprised of five (6 s) condition-specific auditory stimuli. Each of three Aud-MNS runs consisted of eight 30-s blocks (two blocks of each stimulus condition) interleaved with 10-s silent/rest condition blocks (344 s/run). The presentation of stimulus blocks was counterbalanced within and across the three runs, and auditory stimuli were randomized within single blocks. During the scan, as in the training task, participants were asked to focus on a white fixation cross that appeared on a black background in the center of the visual display during auditory stimulus presentation. For 500 ms after each silent/rest condition, a white asterisk symbol was displayed in place of the fixation cross to cue the beginning of a new stimulus block. The primary contrast of interest was action (hand and mouth) sounds *versus* non-action (environmental) sounds, which were previously shown to evoke mirror neuron system activation [48,49].

#### Music-Noise study

The Music-Noise study consisted of two runs, inclusive of four auditory stimulus conditions: single musical notes, single musical chords, human non-word vocalizations, and white noise. Presentation of 12 auditory stimuli, for 2 s each, was randomized within in each stimulus condition block. Within a single run, following the presentation of four consecutive stimulus blocks, one of each condition, was a 24-s silent/rest condition. A white fixation cross was displayed in the center of a black screen during all stimulus and silent/rest conditions. Three presentations of each stimulus block were included in each of two consecutive runs. In preliminary analyses for the Music-Noise study, each sound condition was contrasted with the silent condition. However, since each of the three sound conditions (notes, chords, human vocalizations) elicited similar brain activation patterns, we simplified the primary contrast of interest to sound *versus* silence [43]).

#### Faces study

In the Faces study, participants were asked to passively view two runs of face stimuli consisting of three stimulus conditions: happy, sad, and angry faces. Each of nine stimulus condition blocks contained 10 stimuli presented for 2 s each, followed by a 10-s silent/rest condition. A white fixation cross was displayed in the center of a black screen during all stimulus and silent/rest conditions. Stimuli were randomized within blocks, and blocks were randomized within each run. For the Faces task, the primary contrast of interest was angry faces *versus* fixation, which is known to elicit a very robust fear or anxiety-related response and was previously reported to produce different levels of activation in individuals with WS *versus* typical development [44,50,51].

#### Images study

In the Images study, participants were asked to passively view two runs of ‘Images’ stimuli, which did not include images of humans. Nine blocks, presented in each of two consecutive runs consisted of three stimulus conditions: positive, negative, and neutral images. Each stimulus condition block presented 10 stimuli for 2 s each and was followed by a 10-s silent/rest condition. A white fixation cross was displayed in the center of a black screen during all stimulus and silent/rest conditions. Stimuli were randomized with each run and stimulus condition blocks were balanced across the presentation of the two runs. The primary contrast of interest was negative *versus* neutral images, which was chosen to reveal differences in brain activation related to fear processing or anxiety.

#### Image processing

We performed a series of preprocessing corrections on the fMRI data: slice time correction, 3D motion correction, 3D spatial smoothing (6 mm FWHM Gaussian kernel), and linear trend removal. Data found to exceed 3 mm of translation or 3 degrees of rotation during a single time-series were excluded from the analysis. We registered fMRI images to T1W structural images from the same participant and transformed all images to Talairach space.

To measure changes in BOLD response, a random-effects general linear model (GLM) with separate study (run) predictors was applied to each individual’s fMRI data for each task.

Each of the four extant datasets we used had its own study-specific hypotheses, contrasts, and regions of interest (ROIs), which we included in this *post-hoc* analysis (Sections 2.4.1-4). An additional set of ROIs was specified for analysis across all four studies to test for potential effects of IQ on BOLD activation in 14 primary cognitive regions of the brain [52]. Unilateral ROIs specified for cognitive regions included: anterior cingulate gyrus, cingulate gyrus, posterior cingulate gyrus, superior frontal gyrus, medial frontal gyrus, inferior frontal gyrus, and middle frontal gyrus. All ROIs were defined anatomically using the Talairach-Tournoux Atlas dataset from AFNI (TTatlas+tlrc Dataset; Cox, 1996). A full-list of study-specific and cognitive ROIs can be found in Additional file 2 Table S2. Beta coefficient values for each ROI were derived from study-specific GLM analyses. All image analysis, including data preprocessing, GLM, and ROI analyses, was performed in Brain Voyager QX Software (version 1.9; Brain Innovation, Maastricht, The Netherlands).

#### Analyses

In this study, we aimed to address the interpretation of study-specific ROI activations in the context of study-specific hypotheses. We also investigated whether there were any IQ-related effects in primary cognitive brain regions. In contrast, a whole brain analysis approach would have been more appropriate for a study designed to identify whole brain patterns associated with variability in IQ. To evaluate the relationship between functional activation and intellectual ability, we performed correlation analyses. For each fMRI study, a two-tailed Spearman rank correlation test (IBM SPSS Statistics Software, version 19) was conducted to evaluate the relationship between fMRI contrast-specific β-values, measured for each subject in each ROI, and his or her KBIT-2 scores (composite IQ score, verbal IQ score, non-verbal IQ score).

Using a liberal experiment-wise Type I error rate of 0.01, the Bonferroni-corrected α for each study was given by the number of ROIs within the study (Aud-MNS: 28 ROIs, α = 0.00036; Music-Noise: 24 ROIs, α= 0.00042; Faces: 26 ROIs, α = 0.00038; Images: 26 ROIs, α = 0.00038). These tests were conducted for all participants in a study (‘All Subjects’). Table 3 shows subject enrollment and group mean IQ scores for each of the four studies. In total, we conducted 312 ‘All Subjects’ Spearman rank correlation tests (4 studies × 24–28 brain ROIs per study ×x 3 IQ measures). Since the three IQ measures are intercorrelated, we chose to correct for only one set of IQ measures (Table 3).

**Table 3:** Study enrollment and group mean IQ scores

**fMRI study**	**IQ measure**	**Mean ± SD**	**(*n*)**
**Auditory MNS study**			
	Composite	69.3 ± 20.9	(16)
	Verbal	76.3 ± 18.7	(16)
	Non-verbal	67.6 ±21.6	(16)
**Music-Noise study**			
	Composite	70.1 ± 19.2	(15)
	Verbal	80.4 ± 14.7	(15)
	Non-verbal	66.1 ± 20.6	(15)
**Faces study**			
	Composite	71 ± 15.9	(13)
	Verbal	69.2 ± 20.0	(13)
	Non-verbal	79.2 ± 11.8	(13)
**Images study**			
	Composite	70.5 ± 16.5	(12)
	Verbal	68.3 ± 20.6	(12)
	Non-verbal	79.1 ± 12. 3	(12)

Subject enrollment is shown for each of four fMRI tasks performed. KBIT-2 IQ scores (mean ± SD) are reported for subjects enrolled in each task. The All Subjects group, which includes all participants in a task, was stratified into Low IQ (<1 SD below normal) and High IQ (within 1 SD of normal) subject groups based on scores for each IQ measure given by the KBIT-2: composite, verbal, and non-verbal.

**Table 4:** **fMRI β-value*vs*. IQ rank score correlation analysis results**

**ROI**	**fMRI study**	**IQ measure**	**(*n*)**	ρ*	**Uncorrected *P *value**	**Corrected*P*value**
Right fusiform gyrus	Images	Composite	(12)	0.727	0.007	0.868
		Verbal	(12)	0.734	0.007	0.868
Left insula	Faces	Non-verbal	(13)	0.746	0.003	0.372
Right anterior cingulate	Faces	Composite	(13)	0.728	0.005	0.620
		Non-verbal	(13)	0.735	0.004	0.496
Right inferior frontal gyrus	Faces	Composite	(13)	0.700	0.008	0.992
Right middle frontal gyrus	Music-Noise	Composite	(15)	0.642	0.010	1.00

## Results

### Intellectual assessment

There were no significant differences between verbal and non-verbal IQ scores using a two-tailed Student’s *t*-test. However, consistent with the WS phenotype, the group mean of verbal standard scores was higher than that of non-verbal standard scores.

### Correlation analyses

To assess the effect of IQ on BOLD activation in study-specific regions of interest, we performed correlation analyses. None of the Spearman rank correlation coefficients (ρ) from any of the 312 tests were significant at their respective α level in a two-tailed test. Because our sample size (and therefore our power) in each study was limited, we also wanted to know whether any correlations reached an effect size large enough to be detected in an fMRI study with as many as 26 total subjects (13 per group), which is larger than any of the previously published studies on WS. Such a study would be powered to detect a nominally significant correlation of r=0.58 at an uncorrected *P*<0.01. Of the 312 correlations, seven (5.4%) had effect sizes (ρ) of at least 0.58. These seven correlations were found in five ROIs. In the right fusiform gyrus, β-values from the Images task positively correlated with IQ composite scores (ρ = 0.727, *P* <0.007) and verbal standard scores (ρ = 0.734, *P* <0.007). In the left insula, β-values from the Faces task positively correlated with non-verbal standard scores (ρ = 0.746, *P* <0.003). Also in the Faces study, significant correlations were found in two cognitive ROIs: in the right anterior cingulate, β-values positively correlated with IQ composite (ρ = 0.728, *P* <0.005) and non-verbal standard (ρ = 0.735, *P* <0.004) scores, and in the right inferior frontal gyrus, β-values positively correlated with IQ composite scores (ρ = 0.700, *P* <0.008). Correlation coefficients for each of these tests showed positive correlations between functional activation and intellectual ability. In the right middle frontal gyrus a significant negative correlation was found for IQ Composite (ρ = −0.642, *P* <0.010).

## Discussion

Of the 12 published WS fMRI studies that did not exclude participants based on low IQ, one-third [29,34,36,37] performed a correlation analysis to assess the effect of IQ on BOLD activation in both the WS and control groups. All four of these studies reported no correlation between subject IQ and BOLD activation. In addition, one of the studies that did not exclude participants based on low IQ [39] actually replicated results from two previous studies that did exclude based on IQ [31,42], suggesting IQ does not have an effect on BOLD activation. Likewise, the current study found no significant correlations between IQ and BOLD that survived correction for multiple testing. Additionally, it would be informative to conduct the same analysis in typically-developing individuals.

Each of the fMRI studies we included was designed to target a specific neurocognitive or emotional component of the WS phenotype; however, all involved passive listening and/or viewing of stimuli, with little to no cognitive load. One limitation of these studies is that we did not include eye tracking, button presses, or other attention monitoring strategies to ensure participants were attending to the passive tasks. Some fMRI studies involving a higher cognitive load might elicit brain activation that is negatively correlated with IQ. In this case, including persons with ID in the target sample, but not the control sample, IQ would confound between-group effects. However, it is possible to reduce the cognitive load of some studies without sacrificing construct validity. Future studies should assess the correlation between functional activation and intellectual ability during tasks with higher cognitive load to determine whether participants with ID should be excluded from such studies.

The choice of an appropriate control group and matching criteria is very important and often controversial. For the studies described herein, we were primarily interested in understanding how individuals with WS differ from those with typical development. Given the wide range of intellectual disability in WS, for some fMRI studies that require a higher cognitive load, individuals with other intellectual and developmental disabilities may provide a more appropriate, cognitively matched contrast group to control for potential confounds related to cognitive demand. In future studies, it would also be interesting to investigate these same phenomena in other neurodevelopmental groups with ID, such as Prader-Willi syndrome, autism spectrum disorders, Down syndrome, or Fragile X syndrome, whose neuropsychological profiles are very different from that of people with WS.

## Conclusions

In this study, we aimed to explore the relationship between IQ and BOLD activation using extant fMRI datasets in WS. Using a liberal correction for multiple testing, none of the correlation coefficients from any of the 312 tests were significant, suggesting functional activation was not correlated with intellectual ability across multiple tasks with low cognitive load. Given that exclusion of subjects based on IQ limits the inferences that can be made about the vast majority of individuals with WS, investigators should consider modifications in study design that would still permit investigation of the scientific questions of interest.

Some reports have found neural activity correlates positively with intellectual ability during tasks of higher cognitive load [52,53], while others have not [29,34,36,37]. Graham *et al*. ( [2010]) found evidence that the relationship between IQ and BOLD is complex and depends on multiple factors including, which cognitive processes are employed, which brain region are involved, task complexity and experimental design [54]. Thus, it is important to consider modeling IQ in one’s analysis, even when the IQ among participants is in the normal range. Based on the findings from this study, we propose that investigators measure IQ and, if the outcome of interest is dependent on IQ, it should be controlled for in the analysis. In summary, our findings suggest that it is not necessary to exclude participants with low IQ, especially for low cognitive load tasks, and inclusion of these participants will have the benefit of increased generalization of the findings.

## Abbreviations

Aud-MNS: Auditory mirror neuron system study; BOLD: Blood oxygen level-dependent; fMRI: Functional magnetic resonance imaging; KBIT-2: Kaufman Brief Intelligence Test, Second Edition; ROI: Region of interest; T1W: T1-weighted; WS: Williams syndrome.

## Competing interests

The authors declare that they have no competing interests.

## Authors’ contributions

JRP, SNA, JUB, and TATW conducted the neuroimaging studies. EMD conceived of the study, and TATW primarily designed the study. JRP participated in the design of the study, conducted the analyses, and drafted the manuscript. EMD and TATW helped to draft the manuscript. SNA and JUB edited the manuscript. All authors read and approved the final manuscript.

## Supplementary Material

Additional file 1 **Table S1.** Participant demographics and study enrollment. For each participant, sex, KBIT-2 verbal standard score, non-verbal standard score and IQ composite are reported. An “X” was placed in the appropriate column for each fMRI task in which a participant was enrolled. Click here for file

Additional file 2 **Table S2.** All subjects region of interest correlation coefficients. S.2A. All subjects region of interest correlation coefficients: Auditory Mirror Neuron System fMRI study. S.2B. All subjects region of interest correlation coefficients: Music-Noise fMRI study. S.2C. All subjects region of interest correlation coefficients: Faces fMRI study. S.2D. All subjects region of interest correlation coefficients: Images fMRI study Click here for file
