# Supplementary material for: The effect of intellectual ability on functional activation in a neurodevelopmental disorder: preliminary evidence from multiple fMRI studies in Williams syndrome
Source: J Neurodev Disord. 2012 Oct 26;4(1):24. doi: 10.1186/1866-1955-4-24 (PMC3502608; doi:10.1186/1866-1955-4-24)
Supplement: Additional file 1 — Table S1. Participant demographics and study enrollment. For each participant, sex, KBIT-2 verbal standard score, non-verbal standard score and IQ composite are reported. An “X” was placed in the appropriate column for each fMRI task in which a participant was enrolled. [file 1866-1955-4-24-S1.pdf]

S.1. Participant Demographics and Study Enrollment

ID #	Sex	Verbal	NonVerbal	Composite	Aud-MNS	Music-Noise	Faces	Images
		KBIT-2 Scores			Study Enrollment			
1	F	107	96	102	X	X		
2	F	48	68	53			X	X
3	F	87	76	79	X			
4	F	80	80	77			X	
5	F	71	78	71			X	X
6	M	102	94	98	X	X	X	X
7	M	95	86	89	X	X	X	X
8	M	85	98	90	X		X	X
9	M	77	91	81			X	X
10	F	42	70	51			X	X
11	M	48	68	53			X	X
12	F	48	83	61			X	X
13	M	77	77	73		X	X	X
14	M	84	69	73			X	X
15	F	66	40	48	X	X		
16	M	63	46	53	X	X	X	X
17	F	71	44	52	X	X		
18	M	60	48	49	X	X		
19	M	98	94	96	X	X		
20	M	78	48	55		X		
21	M	81	71	72		X		
22	M	75	68	71	X	X		
23	F	82	61	67		X		
24	M	86	76	78	X	X		
25	M	65	42	48		X		
26	F	62	48	50	X			
27	F	67	54	55	X			
28	M	40	42	40	X			
29	M	57	71	59	X			

Supplemental Table 1. For each participant, sex, KBIT-2 Verbal standard score, Nonverbal standard score and IQ Composite are reported. An "X" was placed in the appropriate column for each fMRI task in which a participant was enrolled.
