# Supplementary material for: The effect of intellectual ability on functional activation in a neurodevelopmental disorder: preliminary evidence from multiple fMRI studies in Williams syndrome
Source: J Neurodev Disord. 2012 Oct 26;4(1):24. doi: 10.1186/1866-1955-4-24 (PMC3502608; doi:10.1186/1866-1955-4-24)
Supplement: Additional file 2 — Table S2. All subjects region of interest correlation coefficients. S.2A. All subjects region of interest correlation coefficients: Auditory Mirror Neuron System fMRI study. S.2B. All subjects region of interest correlation coefficients: Music-Noise fMRI study. S.2C. All subjects region of interest correlation coefficients: Faces fMRI study. S.2D. All subjects region of interest correlation coefficients: Images fMRI study [file 1866-1955-4-24-S2.pdf]

**S.2A.** All Subjects Region of Interest Correlation Coefficients: Auditory Mirror Neuron System fMRI Study

| | | Cognitive ROIs | | | | | | | | | | | | | |
|---|---|---|---|---|---|---|---|---|---|---|---|---|---|---|---|
| | | Superior frontal gyrus | | Medial frontal gyrus | | Middle frontal gyrus | | Inferior frontal gyrus | | Anterior cingulate gyrus | | Cingulate gyrus | | Posterior cingulate gyrus | |
| | | R | L | R | L | R | L | R | L | R | L | R | L | R | L |
| IQ Composite | ρ | .044 | -.131 | -.215 | -.229 | -.026 | -.138 | -.141 | -.218 | -.418 | -.365 | -.026 | -.006 | -.150 | -.065 |
| | p-value | .871 | .629 | .425 | .393 | .922 | .610 | .602 | .418 | .107 | .165 | .922 | .983 | .579 | .812 |
| Verbal IQ | ρ | .065 | -.071 | -.241 | -.206 | .038 | -.112 | -.144 | -.209 | -.468 | -.324 | .006 | .021 | -.212 | -.079 |
| | p-value | .812 | .795 | .368 | .444 | .888 | .680 | .594 | .438 | .068 | .222 | .983 | .940 | .431 | .770 |
| NonVerbal IQ | ρ | .047 | -.044 | -.130 | -.103 | -.027 | -.028 | -.038 | -.087 | -.368 | -.258 | -.113 | .021 | -.065 | .028 |
| | p-value | .862 | .871 | .632 | .704 | .922 | .918 | .888 | .749 | .160 | .335 | .676 | .940 | .811 | .918 |

| | | Task-specific ROIs | | | | | | | | | | | | | |
|---|---|---|---|---|---|---|---|---|---|---|---|---|---|---|---|
| | | Fusiform gyrus | | Precuneus | | Inferior parietal lobe | | Post-central gyrus | | Superior temporal gyrus | | Middle temporal gyrus | | Transverse temporal gyrus | |
| | | R | L | R | L | R | L | R | L | R | L | R | L | R | L |
| IQ Composite | ρ | .047 | -.179 | .450 | .279 | .194 | .097 | -.091 | -.206 | -.012 | -.344 | .082 | -.403 | .103 | -.176 |
| | p-value | .863 | .506 | .080 | .295 | .471 | .721 | .737 | .444 | .966 | .192 | .762 | .122 | .704 | .513 |
| Verbal IQ | ρ | .100 | -.097 | .456 | .344 | .162 | .065 | -.009 | -.032 | -.156 | -.462 | .044 | -.397 | .135 | -.235 |
| | p-value | .713 | .721 | .076 | .192 | .549 | .812 | .974 | .905 | .564 | .072 | .871 | .128 | .617 | .380 |
| NonVerbal IQ | ρ | .065 | -.085 | .416 | .321 | .211 | .187 | -.119 | -.218 | .152 | -.139 | .103 | -.223 | .124 | -.059 |
| | p-value | .811 | .753 | .109 | .225 | .433 | .488 | .660 | .417 | .575 | .609 | .704 | .407 | .648 | .828 |

**S.2B.** All Subjects Region of Interest Correlation Coefficients: Music-Noise fMRI Study

| | | Cognitive ROIs | | | | | | | | | | | | | |
|---|---|---|---|---|---|---|---|---|---|---|---|---|---|---|---|
| | | Superior frontal gyrus | | Medial frontal gyrus | | Middle frontal gyrus | | Inferior frontal gyrus | | Anterior cingulate gyrus | | Cingulate gyrus | | Posterior cingulate gyrus | |
| | | R | L | R | L | R | L | R | L | R | L | R | L | R | L |
| IQ Composite | ρ | -.415 | -.508 | -.200 | -.027 | -.642 | -.390 | -.273 | -.046 | .029 | -.231 | -.100 | -.091 | -.182 | -.063 |
| | p-value | .124 | .053 | .474 | .924 | .010 | .151 | .324 | .869 | .919 | .408 | .723 | .747 | .516 | .825 |
| Verbal IQ | ρ | -.268 | -.461 | -.107 | .068 | -.568 | -.264 | -.143 | .082 | .125 | -.175 | -.018 | -.064 | -.175 | -.125 |
| | p-value | .334 | .084 | .704 | .810 | .027 | .341 | .612 | .771 | .657 | .533 | .950 | .820 | .533 | .657 |
| NonVerbal IQ | ρ | -.451 | -.513 | -.166 | -.088 | -.610 | -.399 | -.208 | -.093 | .045 | -.272 | -.011 | -.005 | -.066 | .027 |
| | p-value | .092 | .050 | .553 | .756 | .016 | .141 | .458 | .742 | .874 | .327 | .970 | .985 | .815 | .924 |

| | | Task-specific ROIs | | | | | | | | | |
|---|---|---|---|---|---|---|---|---|---|---|---|
| | | Cuneus | | Lingual gyrus | | Insular cortex | | Superior temporal gyrus | | Middle temporal gyrus | |
| | | R | L | R | L | R | L | R | L | R | L |
| IQ Composite | ρ | .186 | .130 | .197 | .007 | -.206 | -.088 | .157 | -.032 | -.150 | -.320 |
| | p-value | .507 | .643 | .482 | .980 | .462 | .756 | .576 | .909 | .593 | .245 |
| Verbal IQ | ρ | .218 | .168 | .171 | -.032 | -.182 | -.011 | .011 | -.139 | -.136 | -.377 |
| | p-value | .435 | .550 | .541 | .909 | .516 | .970 | .970 | .621 | .630 | .166 |
| NonVerbal IQ | ρ | .159 | .102 | .204 | -.005 | -.163 | -.025 | .220 | .048 | -.079 | -.270 |
| | p-value | .571 | .718 | .466 | .985 | .562 | .929 | .431 | .864 | .780 | .330 |

**S.2C.** All Subjects Region of Interest Correlation Coefficients: Faces fMRI Study

| | | Cognitive ROIs | | | | | | | | | | | | | |
|---|---|---|---|---|---|---|---|---|---|---|---|---|---|---|---|
| | | Superior frontal gyrus | | Medial frontal gyrus | | Middle frontal gyrus | | Inferior frontal gyrus | | Anterior cingulate gyrus | | Cingulate gyrus | | Posterior cingulate gyrus | |
| | | R | L | R | L | R | L | R | L | R | L | R | L | R | L |
| IQ Composite | ρ | .385 | .321 | .515 | .515 | .385 | .387 | .700 | .481 | .728 | .614 | .512 | .368 | .570 | .224 |
| | p-value | .195 | .285 | .072 | .072 | .195 | .191 | .008 | .096 | .005 | .026 | .074 | .216 | .042 | .462 |
| Verbal IQ | ρ | .285 | .255 | .434 | .418 | .337 | .310 | .600 | .432 | .667 | .562 | .495 | .296 | .454 | .122 |
| | p-value | .345 | .401 | .138 | .156 | .259 | .303 | .030 | .141 | .013 | .046 | .085 | .326 | .119 | .692 |
| NonVerbal IQ | ρ | .426 | .382 | .542 | .517 | .344 | .360 | .635 | .476 | .735 | .663 | .440 | .358 | .443 | .336 |
| | p-value | .146 | .197 | .056 | .070 | .250 | .226 | .020 | .100 | .004 | .014 | .132 | .230 | .130 | .262 |

| | | Task-specific ROIs | | | | | | | | | | | |
|---|---|---|---|---|---|---|---|---|---|---|---|---|---|
| | | Parahippocampal gyrus | | Amygdala | | Cuneus | | Lingual gyrus | | Fusiform gyrus | | Insular cortex | |
| | | R | L | R | L | R | L | R | L | R | L | R | L |
| IQ Composite | ρ | .656 | .382 | .409 | .047 | .152 | -.094 | .205 | .332 | .490 | .365 | .550 | .664 |
| | p-value | .015 | .198 | .165 | .879 | .620 | .760 | .502 | .268 | .089 | .220 | .051 | .013 |
| Verbal IQ | ρ | .523 | .243 | .282 | -.030 | .172 | -.169 | .180 | .210 | .418 | .307 | .429 | .575 |
| | p-value | .067 | .423 | .350 | .921 | .575 | .582 | .557 | .491 | .156 | .307 | .144 | .040 |
| NonVerbal IQ | ρ | .534 | .209 | .248 | -.116 | .151 | .083 | .201 | .520 | .319 | .234 | .619 | .746 |
| | p-value | .060 | .493 | .415 | .707 | .622 | .789 | .511 | .069 | .288 | .442 | .024 | .003 |

**S.2D.** All Subjects Region of Interest Correlation Coefficients: Images fMRI Study

| | | Cognitive ROIs | | | | | | | | | | | | | |
|---|---|---|---|---|---|---|---|---|---|---|---|---|---|---|---|
| | | Superior frontal gyrus | | Medial frontal gyrus | | Middle frontal gyrus | | Inferior frontal gyrus | | Anterior cingulate gyrus | | Cingulate gyrus | | Posterior cingulate gyrus | |
| | | R | L | R | L | R | L | R | L | R | L | R | L | R | L |
| IQ Composite | ρ | .176 | .035 | .317 | .183 | .148 | .109 | .138 | .247 | .000 | .166 | .522 | .282 | .466 | .173 |
| | p-value | .583 | .913 | .315 | .568 | .646 | .735 | .670 | .439 | 1.000 | .607 | .082 | .374 | .127 | .591 |
| Verbal IQ | ρ | .148 | .049 | .293 | .250 | .141 | .123 | .226 | .194 | .046 | .212 | .452 | .176 | .374 | .176 |
| | p-value | .646 | .879 | .356 | .432 | .662 | .702 | .480 | .546 | .887 | .509 | .141 | .583 | .231 | .583 |
| NonVerbal IQ | ρ | .077 | -.084 | .217 | .102 | .011 | -.056 | -.081 | .256 | -.049 | .116 | .420 | .329 | .385 | .245 |
| | p-value | .812 | .795 | .498 | .753 | .974 | .863 | .803 | .422 | .880 | .721 | .174 | .296 | .216 | .442 |

| | | Task-specific ROIs | | | | | | | | | | | |
|---|---|---|---|---|---|---|---|---|---|---|---|---|---|
| | | Parahippocampal gyrus | | Amygdala | | Cuneus | | Lingual gyrus | | Fusiform gyrus | | Insular cortex | |
| | | R | L | R | L | R | L | R | L | R | L | R | L |
| IQ Composite | ρ | .684 | .575 | .212 | .448 | .229 | .028 | .282 | .444 | .727 | .614 | .236 | .212 |
| | p-value | .014 | .050 | .509 | .144 | .473 | .931 | .374 | .148 | .007 | .034 | .460 | .509 |
| Verbal IQ | ρ | .642 | .582 | .286 | .515 | .198 | -.007 | .236 | .363 | .734 | .561 | .215 | .183 |
| | p-value | .024 | .047 | .368 | .087 | .538 | .983 | .460 | .246 | .007 | .058 | .502 | .568 |
| NonVerbal IQ | ρ | .508 | .371 | .011 | .228 | .165 | -.095 | .189 | .385 | .602 | .543 | .186 | .123 |
| | p-value | .092 | .235 | .974 | .477 | .609 | .770 | .556 | .216 | .038 | .068 | .564 | .704 |
